# Pneumolysin and the bacterial capsule of *Streptococcus pneumoniae* cooperatively inhibit taxis and motility of microglia

**DOI:** 10.1186/s12974-019-1491-7

**Published:** 2019-05-18

**Authors:** Sabrina Hupp, Denis Grandgirard, Timothy J. Mitchell, Stephen L. Leib, Lucy J. Hathaway, Asparouh I. Iliev

**Affiliations:** 1 Institute of Anatomy, University of Bern, Baltzerstrasse 2, 3012 Bern, Switzerland; 2 Institute for Infectious Diseases, University of Bern, Friedbühlstrasse 51, 3010 Bern, Switzerland; 3 Institute of Microbiology and Infection, College of Medical and Dental Sciences, Biosciences Building, University of Birmingham, Edgbaston, Birmingham, B15 2TT UK

**Keywords:** Microglia, Bacterial capsule, Pneumolysin, *Streptococcus pneumoniae*, Meningitis

## Abstract

**Background:**

*Streptococcus pneumoniae* is the cause of a highly lethal form of meningitis in humans. Microglial cells in the brain represent the first line of defense against pathogens, and they participate in the inflammatory response. The cholesterol-dependent cytolysin pneumolysin and the bacterial capsule are key pathogenic factors, known to exacerbate the course of pneumococcal meningitis.

**Methods:**

We utilized live imaging and immunostaining of glial cells in dissociated and acute brain slice cultures to study the effect of pneumococcal factors, including the cholesterol-dependent cytolysin pneumolysin and the pneumococcal capsule, on microglial motility and taxis.

**Results:**

In brain tissue, primary microglia cells showed an enhanced response towards lysates from bacteria lacking capsules and pneumolysin as they moved rapidly to areas with an abundance of bacterial factors. The presence of bacterial capsules and pneumolysin cumulatively inhibited microglial taxis. In mixed cultures of astrocytes and microglia, the motility of microglia was inhibited by capsular components within minutes after exposure. The reduced motility was partially reversed by mannan, a mannose receptor inhibitor. The effects on microglia were not mediated by astrocytes because pure microglial cells responded to various pneumococcal lysates similarly with distinct cell shape changes as seen in mixed cultures.

**Conclusions:**

Our data indicate that microglia possess the capacity for a very agile response towards bacterial pathogens, but key pathogenic factors, such as pneumococcal capsules and pneumolysin, inhibited this response shortly after a bacterial challenge. Furthermore, we demonstrate for the first time that the bacterial capsule affects cellular behaviors such as motility and taxis.

**Electronic supplementary material:**

The online version of this article (10.1186/s12974-019-1491-7) contains supplementary material, which is available to authorized users.

## Background

Bacterial meningitis is a disease with high lethality and substantial disability of surviving patients [1]. While antibiotic therapy has improved the outcome substantially, lethality, especially by some more pathogenic strains (e.g., the endemic ST217 virulent clone in the African meningitis belt), still remains very high, exceeding 60% in some parts of the world [2]. The most frequent pathogen causing meningitis worldwide is *S. pneumoniae* (pneumococcus) [3]. Experiments in meningitis animal models infected with *S. pneumoniae* indicates that the cholesterol-dependent cytolysin, pneumolysin (PLY), and the bacterial cell capsule play critical roles in the pathogenesis of the disease, as bacteria deficient in either of these factors are not as pathogenic as the wild-type strains [4–7]. Nevertheless, some differences between PLY-deficient and bacterial capsule-deficient mutants have been observed; for example, capsule mutants are rapidly eliminated, while the PLY-deficient strains still cause meningitis but with a greatly reduced lethality and a milder disease course.

PLY exercises multiple pathogenic effects in the brain tissue, such as astrocyte shape changes, increased bacterial tissue penetration, and glutamate-mediated synaptic changes through astrocytes [8, 9]. These effects are initiated by calcium influx, actin, and tubulin reorganization as well as small GTPase activation, and they occur at nonlytic concentrations of the toxin—concentrations that have no lytic effects in tissues and minimal (< 5%) lysis in culture [10–13]. Apart from the pro-inflammatory effects of PLY on microglia [14], the migratory behavior of this highly motile surveillance phagocyte of the brain in the presence of pneumococcal infection remains unknown. While microglia are intimately involved in neuroinflammatory neuronal damage and neuroprotection [15, 16], their motility within the brain remains relatively unknown in the context of brain infections.

Bacterial capsules have been extensively studied in the context of bacterial virulence. The capsule plays a role in the colonization of the nasopharynx and the penetration of bacterial pathogens through mucous barriers and yields protection against opsonization/phagocytosis by host immune cells during bacteremia [17, 18]. In meningitis models, the presence of capsule does not inhibit phagocytosis by microglia but improves intracellular survival of the pathogen [7].

In this work, we examined the effect of *Streptococcus pneumoniae* and its major pathogenic factors (the capsule and PLY) on microglial tissue migratory behavior in acute brain slices and in primary cultures.

## Methods

### PLY preparation

Wild-type PLY was expressed in *Escherichia coli* BL-21 cells (Stratagene, Cambridge, UK) and purified via metal affinity chromatography. The purified PLY was tested for the presence of contaminating Gram-negative LPS using the colorimetric LAL (Limulus amebocyte lysate) assay (KQCL-BioWhittaker, Lonza, Basel, Switzerland). All purified proteins showed < 0.6 endotoxin units/μg of protein. Hemolytic activity was judged by a standard assay described previously [19]. Briefly, one hemolytic unit (HU) was defined as the minimum amount of toxin needed to lyse 90% of 1% human erythrocytes per milliliter within 1 h at 37 °C. Equivalent lytic capacity in red blood cells does not explicitly correspond to equivalent lytic capacity in other cell types. For PLY, we determined the hemolytic capacity of 40,000 HU/mg.

### Bacterial cultures

A wild-type D39 strain, PLY-deficient mutant (ΔPLY), was kindly provided by Jeremy Brown (University College London, UK) and James Paton (University of Adelaide, Australia). Additionally, capsule-deficient mutant (ΔCps) [20] and double mutant strains (ΔPLYΔCps) [21] (all mutants produced in D39 background) of *S. pneumoniae* were used. Clinical pneumococcal isolate of strain 106.66 with a capsule serotype 6B and its capsule knock out mutant 106.66 Janus (nonencapsulated) were used in some experiments [20]. The strains were plated on blood agar plates (Columbia agar with 5% sheep blood; Oxoid Limited, Hampshire, UK) and incubated at 37 °C under anaerobic conditions overnight. Single colonies were selected and grown to the mid/late log phase in brain heart infusion broth (BHI; Becton Dickinson and Company, le Pont de Claix, France). At this point, cultures were centrifuged, pellets were washed three times in phosphate-buffered saline (PBS), and serial dilutions were plated on blood agar plates to determine the colony-forming units (CFU). The bacteria were finally resuspended in PBS and incubated overnight with 10 μg/ml ceftriaxone (Sigma-Aldrich Chemie GmbH, Schnelldorf, Germany) to produce lysates. Then, the lysates were frozen at − 80 °C for at least one night. In all experiments, lysates equivalent to 20 million CFU/ml (cell culture) or 70 million CFU/ml (acute brain slices) were used.

### Eukaryotic cell cultures and culture treatments

Primary mixed cultures of mouse astrocytes and microglia (also referred to as mixed glial cell cultures) were prepared according to a well-established protocol from the cortices of newborn C57 BL/6 mice (postnatal day 3; Janvier Labs, Le Genest-Saint-Isle, France) in Dulbecco’s-modified Eagle’s medium (high glutamate) (Thermo Fisher Scientific, Waltham, MA, USA). The growth medium was supplemented with 10% fetal bovine serum (PAN Biotech, Aidenbach, Germany) and 1% penicillin/streptomycin (Thermo Fisher Scientific). Fourteen days after preparation and seeding in 75 cm^2^ cell culture flasks (TPP, Trasadingen, Switzerland), the cells were harvested. Culture treatments with bacterial lysates were performed in serum-free medium to avoid PLY inactivation by serum cholesterol. The isolation of pure microglia was achieved utilizing their high adhesive properties. Briefly, after mild trypsinization (0.05% Trypsin-EDTA (ethylenediaminetetraacetic acid) (Thermo Fisher Scientific)), the mixed glial cell suspension was incubated for 45 min at 37 °C in a culture dish to allow adhesion before all other cell types were removed. Then, dishes were thoroughly washed, yielding a > 98% pure microglial culture (verified by ísolectin B4 immunofluorescence (Additional file 1: Figure S1)).

Acute brain slices were prepared from infant (postnatal day 10–14) C57 Bl/6 mice via decapitation and vibratome sectioning (350 μm) (Leica VT1000S, Leica Biosystems, Wetzlar, Germany) in artificial cerebrospinal fluid (aCSF) (in mM—119 NaCl, 2.5 KCl, 1.2 NaH_2_PO_4_, 25 NaHCO_3_, 12.5 glucose, 2 MgSO_4_, 2 CaCl_2_, pH = 7.3–7.4) continuously oxygenized with carbogen gas (95% O_2_, 5% CO_2_) at 4 °C. The slices were allowed to adapt in carbonated aCSF with 1% penicillin/streptavidin (Thermo Fisher Scientific) and 1% glucose (Carl Roth) at 33 °C for 30 min before being challenged with lysates in the same buffer (pH = 7.3) at 37 °C for 6 to 12 h. In these acute slices, cell lysis never exceeded 5% within 12 h (as determined by an LDH assay (Promega, Duebendorf, Switzerland)). In some experiments, cultures were treated with mannan from *Saccharomyces cerevisiae* (Sigma-Aldrich), CpG-DNA (TIB Molbiol, Berlin, Germany), or R406 (inhibitor of Syk; BioVision Biotech Inc., Milpitas, CA, USA).

### Immunohisto- and immunocytochemistry

Acute brain slices were fixed with 1.5% formalin (Sigma-Aldrich) solution for 2 h. After overnight permeabilization in 0.3% Triton X-100 (Carl Roth) in PBS, the slices were blocked in 4% BSA (bovine serum albumin)/PBS (Carl Roth) for 2 h and incubated overnight at room temperature with anti-Iba1 rabbit antibody (1:200; Abcam, Cambridge, UK) and with secondary antibody goat anti-rabbit tagged with Cy3 (1∶200 dilution; Dianova GmbH, Hamburg, Germany) for 2 h at room temperature. Glial cell cultures were fixed with 4% formalin, followed by 0.1% Triton X-100 on ice for 5 min, and blocked with 4% BSA/PBS for 30 min and rabbit anti-phospho-Syk Tyr525/Tyr526 (pSyk) (1:500 dilution; Cell Signaling Technology, Danvers, MA, USA), isolectin B4-Alexa568 (1:500 dilution; Thermo Fischer Scientific), and Iba1 antibodies. Then the samples were incubated with goat anti-rabbit secondary antibody tagged with Cy3 (1∶200 dilution; Dianova). In some experiments, cellular F-actin was stained with phalloidin-Alexa488 (1:200, Thermo Fischer Scientific). All samples were preserved with ProLong Gold antifade reagent (Thermo Fischer Scientific).

Quantification of the immunocytochemistry samples was performed after confocal imaging with defined constant device settings using × 63 oil objective. The average intensities measured within the border of individual cells (excluding the area of the nucleus) were pooled for each experimental group.

### Bead phagocytosis assay

Endocytosis was tested using 1 μm fluorescent yellow-green carboxylate-modified polystyrene latex beads (Sigma-Aldrich) coated with ovalbumin. Briefly, after being washed in MES (2-(N-morpholino)ethanesulfonic acid) buffer (pH = 6.1) (Sigma-Aldrich), the beads were suspended to give a 1% suspension and incubated with 4 mg/ml ovalbumin (Sigma-Aldrich) in MES overnight with gentle shaking following the manufacturer’s instructions. Thereafter, the beads were washed in PBS and stored as a 1% suspension. Cells pre-treated with bacterial lysates were incubated with bacterial lysates for 90 min in serum-free medium before adding beads, dissolved at 1:5000 in the medium. Cells were fixed 60 min later with 4% formalin and stained with rabbit anti-ovalbumin antibody (1:500 dilution; Sigma-Aldrich) without membrane permeabilization (thus staining only extracellular beads) and goat anti-rabbit Cy3 secondary antibody as described above. Phagocytosed beads showed green fluorescence (their native fluorescence), but were anti-ovalbumin-negative. Extracellular beads were both fluorescently green and red (Cy3 ovalbumin staining).

### Live imaging and conventional microscopy

Cells were incubated in Leibovitz’s CO_2_-independent cell culture medium (Thermo Fisher Scientific) in PLO (poly-l-ornithine)-coated borosilicate coverslip-bottom dishes (Sarstedt, Nuembrecht, Germany) [12]. Primary mouse glial cultures were visualized on an Olympus Cell^M imaging system (Olympus Deutschland GmbH, Hamburg, Germany) at a temperature of 37 °C with a combination of a heating plate and custom-built microscope incubator with a heater and thermostat feedback loop, using × 10 or × 20 dry objectives. As a time point 0, we indicated always the first image after the challenge with vehicle or bacteria. Thus, there was always a minimal gap between treatment and beginning of imaging. The live image series were saved in a tiff format, visualized on ImageJ, and traced manually with manual tracing plugin from the microscopy plugin collection of Tony Collins (McMaster Biophotonics Facility, McMaster University, Ontario, Canada). PI (propidium iodide) and DAPI (4,6-Diamidin-2-phenylindol) stains (Thermo Fisher Scientific) were used at working concentrations of 1 μg/ml. High-resolution transmission and fluorescence images were acquired on a Zeiss LSM 880 (Carl Zeiss AG, Oberkochen, Germany) using × 63 oil immersion objectives and an optical zoom between 2 and 4 with laser wavelengths of 488 and 567 nm, corresponding to the excitations of the respective fluorophores. In live imaging experiments, the morphology and taxis of cells were analyzed using software-assisted tracing by ImageJ software (ver. 1.57, NIH, Bethesda, USA).

### Enzyme-linked immunosorbent assay (ELISA)

Acute slices were incubated for 6 h with vehicle or D39 lysates and transferred to 0.3% Triton X-100 in PBS, supplemented with cOmplete mini EDTA-free protease inhibitor cocktail tablets (Roche Diagnostics, Mannheim, Germany). Subsequently, the samples were shock frozen in liquid nitrogen, thawed, homogenized, and left on ice for 1 h. After centrifugation at 15,000×*g* for 10 min, the supernatant was collected and further analyzed for MIF (macrophage migration inhibitory factor) using an ELISA kit according to manufacturer’s instructions (BioLegend Inc., San Diego, USA).

### Statistical analysis

Statistical analysis was performed using GraphPad Prism 4.02 for Windows (GraphPad Software Inc., La Jolla, CA, USA). The statistical tests included Mann-Whitney *U* tests (comparing two groups differing in one parameter) or a Wilcoxon matched pairs test.

## Results

**Fig. 1:**
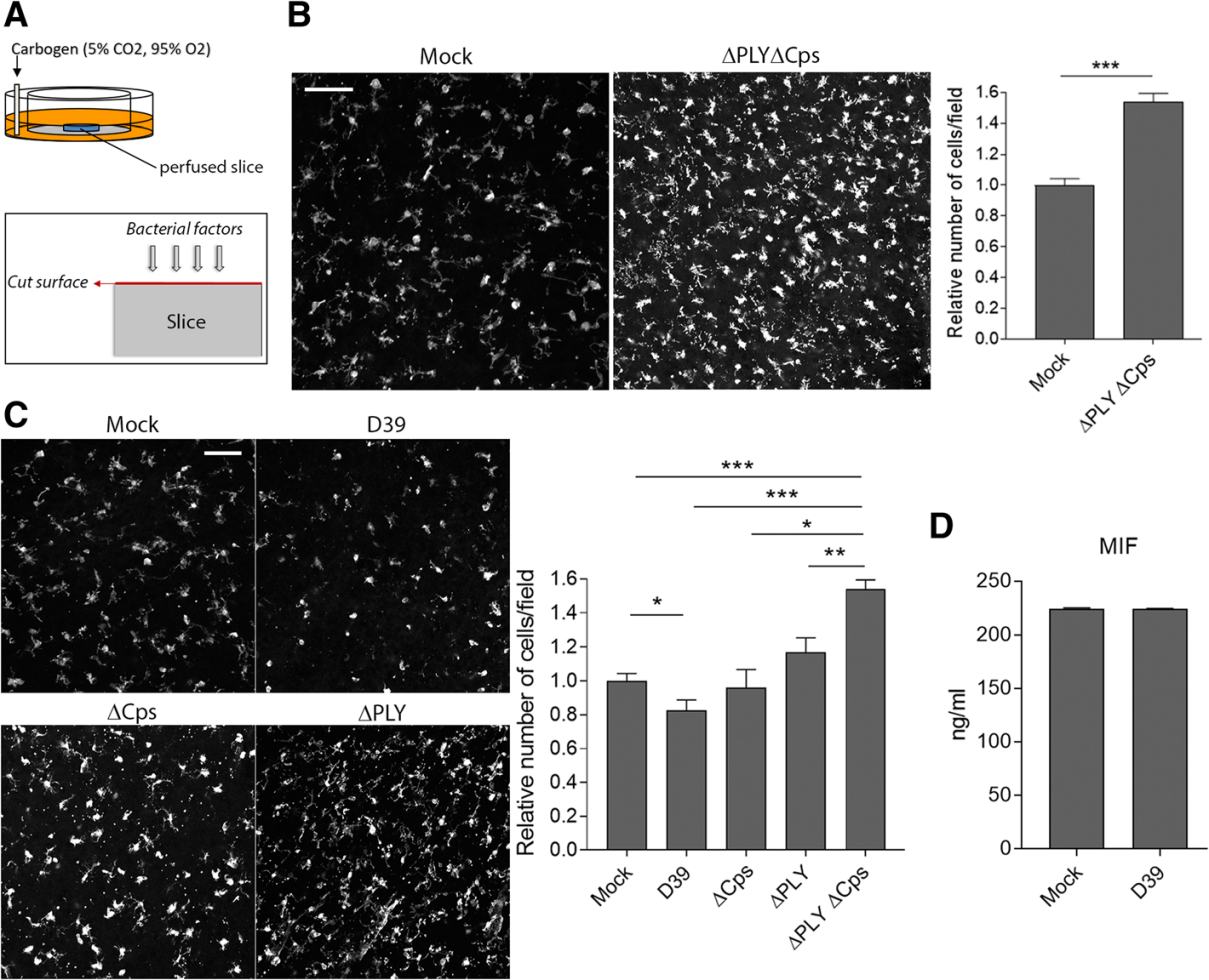
Affected microglial taxis in acute brain slices by capsules and PLY. **a** Schematic diagram of the acute brain slice perfusion system (top) and the cut surface interface, where the taxis of Iba1-stained microglia was analyzed (bottom). **b** The number of microglia recruited to the surface of the slices was significantly greater in slices exposed to bacterial lysates from double mutant (PLY and capsule-deficient (∆PLY∆Cps)) bacteria versus mock-treated slices. **c** Significant reduction in the substantial taxis of microglial cells towards the slice surface in the ∆PLY∆Cps group once PLY expression (∆Cps), capsule expression (∆PLY), or both (PLY and capsule) expressions (D39) were restored. Scale bars, 30 μm. **d** Similar protein levels of MIF in homogenates from acute brain slices 6 h after mock challenge or exposure to D39 bacterial lysates. All values represent the mean ± SEM, *n* = 8 slices from six animals in three experimental series (**a**–**c**), *n* = 6 slices (**d**). *p < 0.05, **p < 0.01, ***p < 0.001. In all bacterial lysis experiments, the lysate concentration was 70 million CFU/ml

Acute brain slices represent a suitable system for the analysis of microglial behavior in areas of damage, such as the cutting interface [22]. Similar to meningitis, where cortical necrosis occurs, the cutting interface contains multiple damaged cells that initiate a chemotactical response from microglia through the release of ATP (adenosine triphosphate) and glutamate (Fig. 1a, Additional file 2: Figure S2) [23, 24]. We prepared acute brain slices from infant C57 Bl/6 mice after 30 min of conditioning in oxygenated aCSF following a standard protocol [25]. We exposed them to 70 million CFU equivalent/ml lysate from low pathogenic nonencapsulated pneumococcal mutants without PLY and capsule (∆PLY∆Cps) for another 6 h. Staining with Iba1 demonstrated that microglia rapidly clustered along the brain slice cut interface after sensing bacterial lytic products (Fig. 1b; 3D reconstruction is shown in Additional file 3: Movie M1 and Additional file 4: Movie M2). However, for bacteria that expressed either PLY or the capsule, this effect was inhibited (Fig. 1c). When slices were exposed to wild-type D39 lysates (containing both the capsule and PLY), microglial taxis was significantly inhibited compared to mock-treated slices (Fig. 1c, 3d reconstruction is shown in Additional file 5: Movie M3). We did not observe elevated lytic cell death on the surface of D39-treated slices (propidium iodide staining) versus mock-treated slices (not shown), indicating that none of our findings were due to acute cell death. Analysis of the protein levels of MIF, which can inhibit microglia migration, did not demonstrate any difference between homogenates of mock-treated and D39-treated acute brain slices (Fig. 1d).

**Fig. 2:**
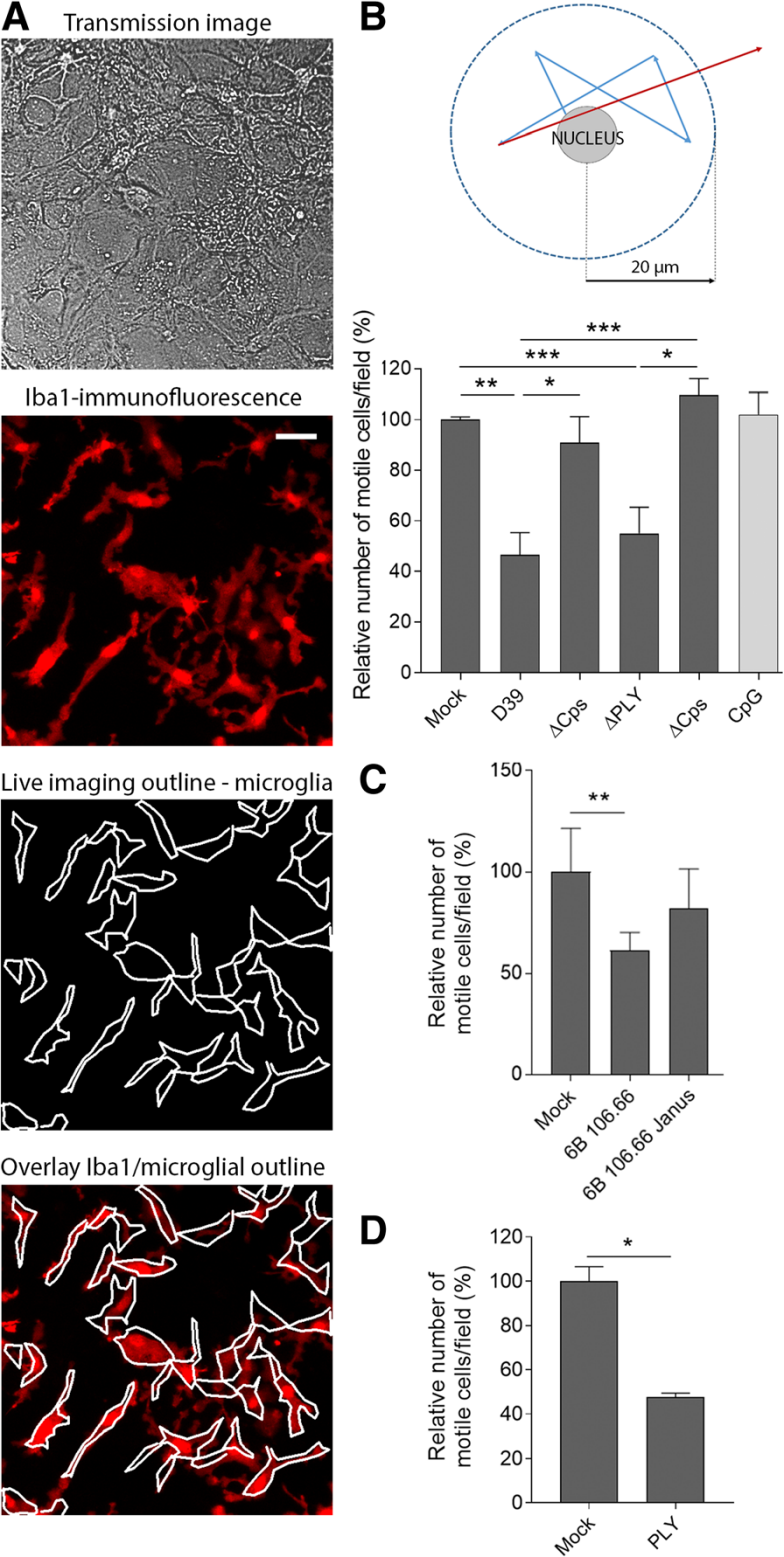
Inhibition of microglial motility by pneumococcal components. **a** Identification of microglia (vs. astrocytes) in mixed glial cultures based on microglial motility in transmission images (see supplementary movies). Compared with a transmission image, microglial outlines after live imaging and Iba1-immunofluorescent imaging of the same area after fixation are shown, demonstrating correct identification of all microglial cells in the imaging field. Scale bar, 40 μm. **b** Determination of basic microglial motility from live imaging and a count of cells with displacements greater than 20 μm. The presence of a bacterial capsule was associated with significantly less motile microglia in the D39 strain background. For comparison, 1 μM CpG-DNA (a pro-inflammatory TLR9 agonist) was also included. **c** Similar trend of motility inhibition was observed in the clinical 106.66 isolate background (106.66 (capsule serotype 6B), 106.66 Janus (capsule-deficient mutant)). **d** Challenge of glial cells with 0.1 μg/ml PLY inhibited microglial motility. All values represent the mean ± SEM, *n* = 5 independent experiments, *p < 0.05, **p < 0.01, ***p < 0.001. In all bacterial lysis experiments, the lysate concentration was 20 million CFU/ml

Next, we analyzed the motility of microglia in mixed glial cultures. The differentiation between directed taxis and general motility is important for the analysis of cell behavior. While directed taxis represents a microglial directional response and recruitment towards areas, where chemotactic factors are released, general motility characterizes the ability of microglia to migrate with specific velocity through the tissue (motility). Normally, after 14 days of dissociated culturing, astrocytes form a monolayer and some microglia float over the surface of the monolayer, while most of them move actively among astrocytes. This behavior is different from the directed taxis towards the cut surface. To verify that we could reliably track microglia by live imaging, we applied immunocytochemistry to confirm the specificity of our live imaging microglial identification approach (Fig. 2a, Additional file 6: Movie M4 and Additional file 7: Movie M5). The overlap was nearly complete. Next, we analyzed the proportion of microglial cells moving beyond a radius of 20 μm (the diameter of an adherent microglial cell, assuming as a center of the field the nucleus of microglia in the first image) in the dish and found that the bacterial capsule was the critical factor that inhibited microglial motility (Fig. 2b). Similarly, the clinical nasopharyngeal isolate 106.66 (capsule serotype 6B) strain of *S. pneumoniae* inhibited microglial motility, while the nonencapsulated 106.66 Janus mutant failed to affect significantly microglial motility (Fig. 2c). The lysis of glial cells in any of the treatment groups did not exceed 5% (minimal lysis was observed only in the cultures treated with PLY-containing lysates), and none of the analyzed cells were PI-positive, confirming that the observed effects were not due to lytic cell death. Surprisingly, exposure of mixed glial cells to sublytic amount of recombinant PLY (0.1 μm/ml) inhibited microglia taxis, which was in contrast with the data with the knockout bacteria treatment (Fig. 2d).

**Fig. 3:**
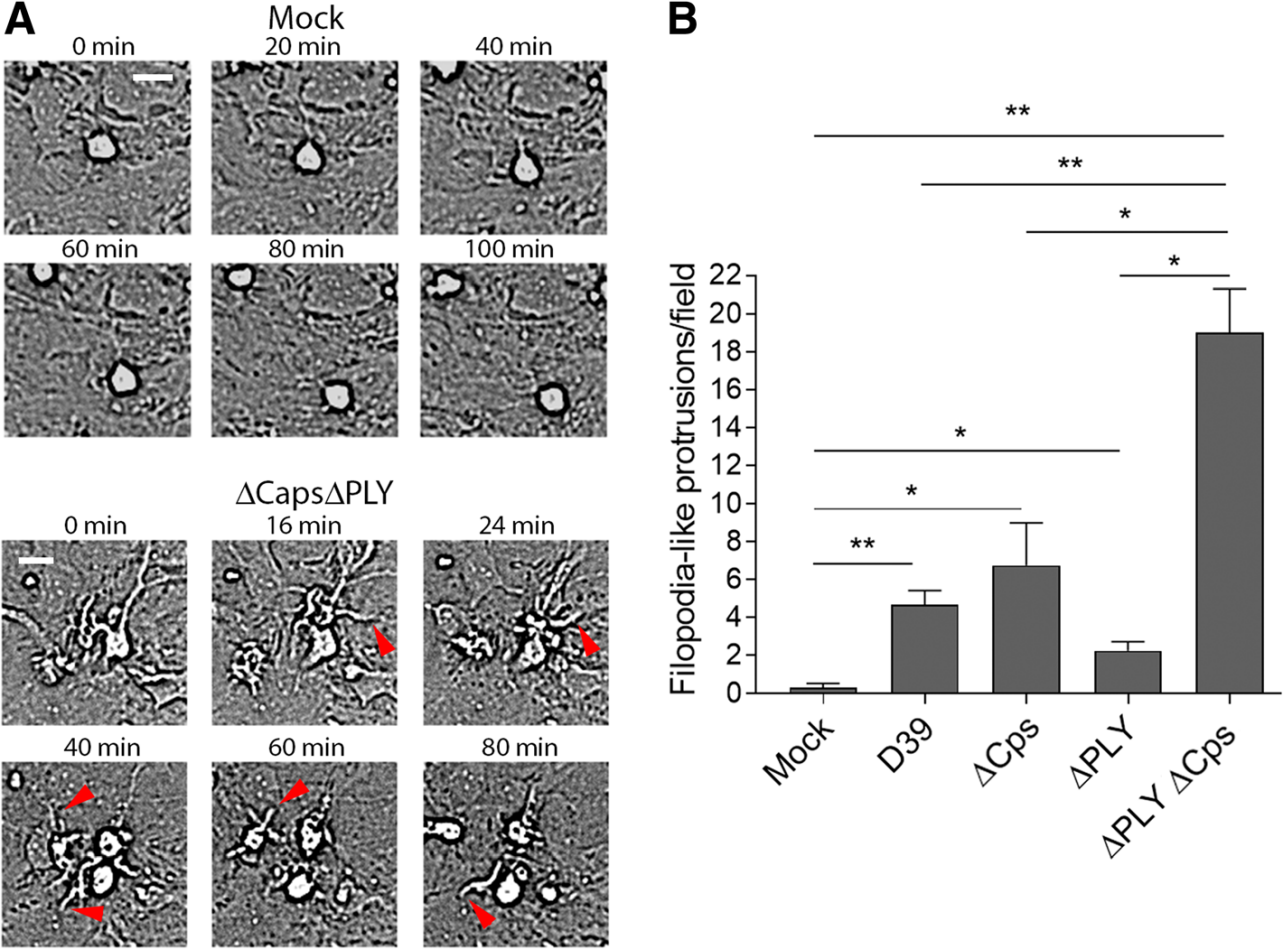
Enhanced filopodia-like process formation by pneumococcal components. **a** Images of mock-treated and ∆PLY∆Cps-treated mixed glial cultures at the indicated time points, demonstrating the lack of filopodia-like membrane extensions in the mock-treated cells and multiple long and dynamic filopodia-like tentacles in the double mutant-treated cells (red arrows). Scale bars, 20 μm. **b** Statistical analysis of the filopodia-like process formation dynamics, demonstrating the inhibition of their formation in the presence of bacterial capsules or PLY compared to double mutant-treated cells. All values represent the mean ± SEM, *n* = 5 independent experiments, *p < 0.05, **p < 0.01. In all bacterial lysis experiments, the lysate concentration was 20 million CFU/ml

When exposed to double pneumococcal mutants (∆PLY∆Cps), microglia in mixed glial cultures formed multiple motile and very long filopodia-like tentacles (processes with length beyond 10 μm, extending above the monolayer of astrocytes). In mock-treated cultures, such processes with length under 10 μm were rarely observed (Fig. 3a, Additional file 8: Movie M6). The formation of these tentacles was significantly attenuated when PLY and/or the capsules were present, but still, these processes were more often observed in microglia treated with bacteria than in the mock control (Fig. 3b).

**Fig. 4:**
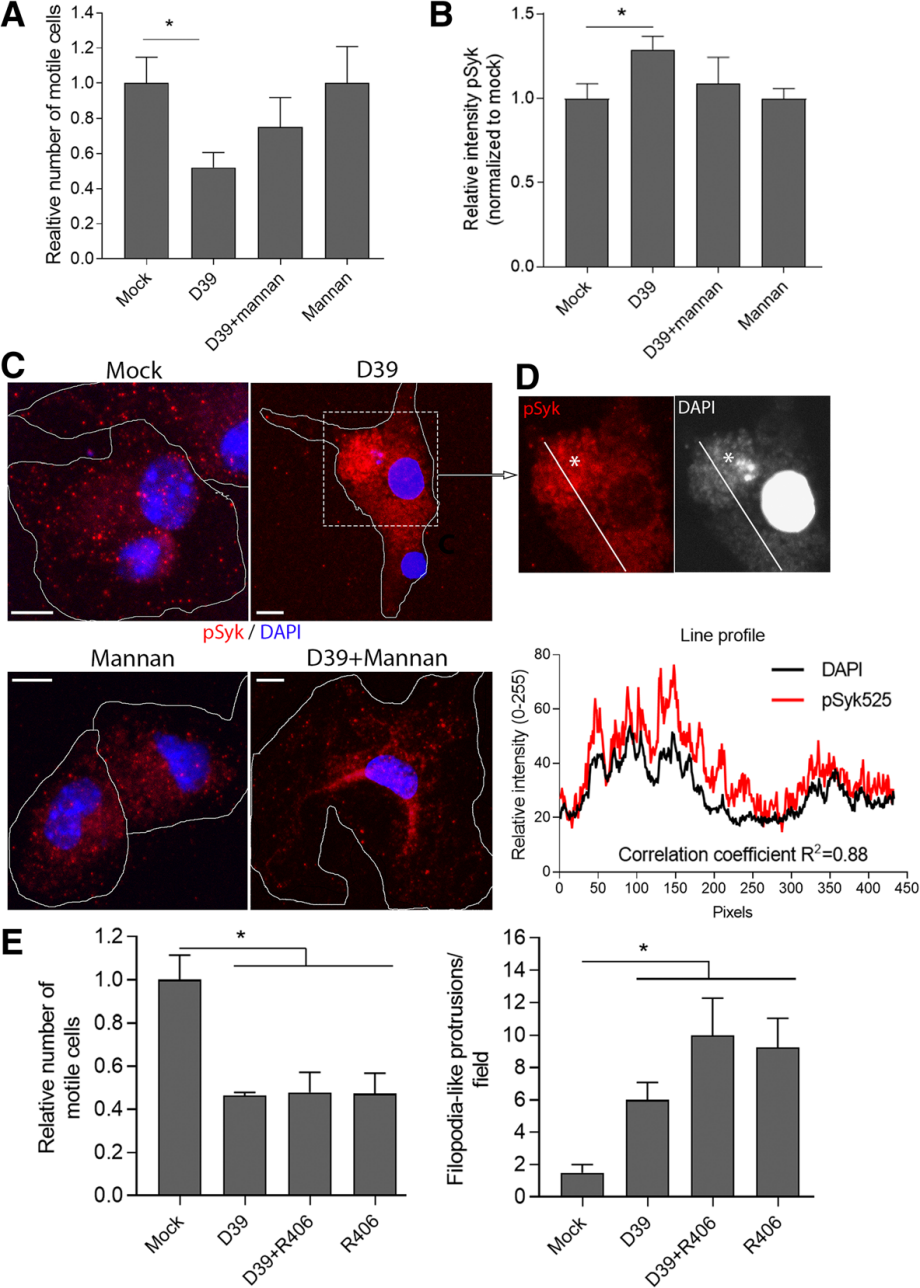
Involvement of pSyk in the motility changes. **a** Pretreatment with 2.5 mM mannan diminished the inhibitory effect of D39 lysates on microglial motility in mixed glial cultures. All values represent the mean ± SEM, *n* = 4 independent experiments, *p < 0.05. **b** Increased Syk kinase phosphorylation (at positions Tyr525 and Tyr526) in microglial isolates after exposure to D39 bacterial lysate for 120 min was diminished by pretreatment with mannan. All values represent the mean ± SEM, *n* = 20–25 cells, *p < 0.05. **c** Sub-cellular distribution of phospho-Syk before and after exposure to D39 bacterial lysates in the presence and absence of 2.5 mM mannan. Microglial exposure to D39 lysates produced clustered pSyk staining around DAPI-positive areas outside the nucleus, most probably containing DNA-rich bacterial products. This clustering was fully reverted by mannan. **d** Enlarged fragment of the D39-treated microglia image in **c**, demonstrating practically a complete overlap (correlation coefficient *R*^2^ = 0.88) between the pSyk accumulation and the DAPI staining of the cytosol. The asterisk indicates a large round DAPI-enriched cytosolic structure, resembling multivesicluar body. Scale bars, 10 μm. **e** Exposure of mixed glial cultures to 500 nM R406 Syk-inhibitor inhibited microglial motility and enhanced filopodia formation similarly to the exposure of the cells to D39 lysates. All values represent the mean ± SEM, *n* = 4 independent experiments, *p < 0.05. In all bacterial lysis experiments, the lysate concentration was 20 million CFU/ml

Bacterial capsules can activate C-type lectin receptors (CLRs) and, more specifically, the mannose receptor. The latter can be inhibited by D-mannose and mannan treatment [26]. Therefore, we pretreated glial cells with 2.5 mM mannan for 1 h and exposed them to D39 lysates. There was a partial reversal of motility inhibition (Fig. 4a). We also analyzed the phosphorylation of the spleen tyrosine kinase (Syk) (a secondary messenger of several CLRs and the mannose receptor [27]) at positions Tyr525/Tyr526 (further referred as pSyk), which was slightly, but significantly, elevated by exposure to D39 lysates (as determined by quantitative immunocytochemical fluorescent staining) and inhibited by mannan (Fig. 4b). Immunocytochemistry for pSyk showed a staining in multiple tiny vesicular structures throughout the cytosol (Fig. 4c). When exposed to D39 lysates, however, the pSyk staining changed its distribution and clustered completely around compartments, containing DAPI-positive fluorescence outside the microglial nucleus. This cytosolic DAPI staining was observed only when bacterial lysates were present, most probably corresponding to endo-/phagocytosed bacterial products and bacterial DNA (Fig. 4c, d). We excluded the possibility these DAPI accumulations could be micronuclei, since the latter requires at least one divisional cycle to form and do not occur so massively within such short time frame in a cell type that has relatively low divisional rate [28]. Treatment with mannan restored the pSyk staining as seen in the mock-treated cells (Fig. 4c). The phosphorylation of Syk at Tyr525/Tyr526 or its recruitment towards specific compartments does not tell us about the level of activation. Therefore, we tested the effect of a selective Syk inhibitor, R406, on microglial motility. Surprisingly, R406 inhibited microglial motility and initiated the formation of filopodia-like tentacles, creating a phenotype, strikingly resembling the effect of D39 lysates on microglia (Fig. 4e).

**Fig. 5:**
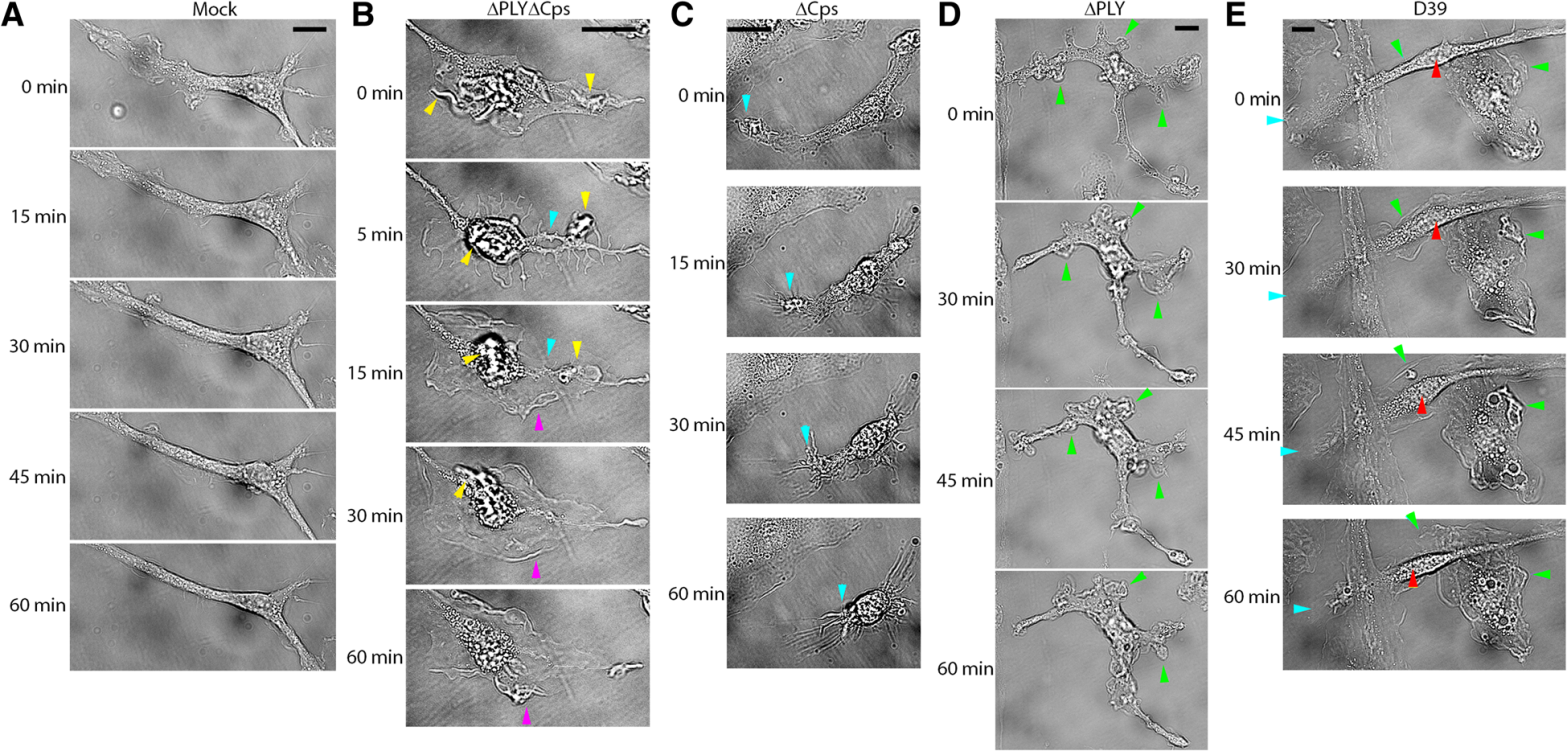
Live imaging patterns of the altered microglia morphology by pneumococcal components. **a** Live imaging of primary microglia, demonstrating normal membrane dynamics and minimal cell motility. **b** Very dynamic membrane changes in microglia (yellow arrows indicate rapidly formed filopodia-like membrane extensions above the level of the focal plane, and cyan and magenta arrows indicate membrane shrinking and expansion respectively). **c** Expression of PLY and lack of bacterial capsule led to substantial cell shrinkage (cyan arrow indicates the shrinking front of the cell) with the formation of multiple post-retracted pseudofilopodia. **d** Lack of PLY but the presence of bacterial capsule in lysates produced very dynamic membrane changes with multiple tiny ruffles that appeared and vanished rapidly (green arrows). **e** Wild-type (D39) bacterial lysates produced a combination of cell shrinkage and displacement (cyan and red arrows respectively) and multiple ruffles (green arrows) in exposed microglial cells. All scale bars, 20 μm. In all bacterial lysis experiments, the lysate concentration was 20 million CFU/ml

To confirm that all microglial changes were independent of the presence of astrocytes or the release of active factors from them, we isolated pure microglia (see the “Methods” section) and exposed them to bacterial lysates. Mock-treated microglia demonstrated multiple small wave-like ruffles and some dynamic physiological membrane changes (Fig. 5a, Additional file 9: Movie M7). Bacteria with knocked out capsules and PLY (∆PLY∆Cps) induced long filopodia-like membrane extensions above the cell level and massive ruffles in microglia (Fig. 5b, Additional file 10: Movie M8), demonstrating clearly increased membrane dynamics compared to those of mock-treated cells. When PLY expression in bacteria was restored (∆Cps), cells predominantly shrunk (Fig. 5c, Additional file 11: Movie M9). When the capsules of bacteria were restored (∆PLY bacteria), multiple motile membrane ruffles, much more dynamic and non-synchronized than those in the double mutant bacteria-treated microglia, were observed (Fig. 5d, Additional file 12: Movie M10). Finally, exposure to the wild-type D39 strain-initiated shrinkage of the cell body and enlargement and synchronization of the wave-like ruffles towards the center of the cells (Fig. 5e, Additional file 13: Movie M11). These findings confirmed that microglia respond to different pneumococcal mutants rapidly and in a distinct manner in the absence of astrocytes, and these effects were consistent with our findings in mixed glial cultures.

**Fig. 6:**
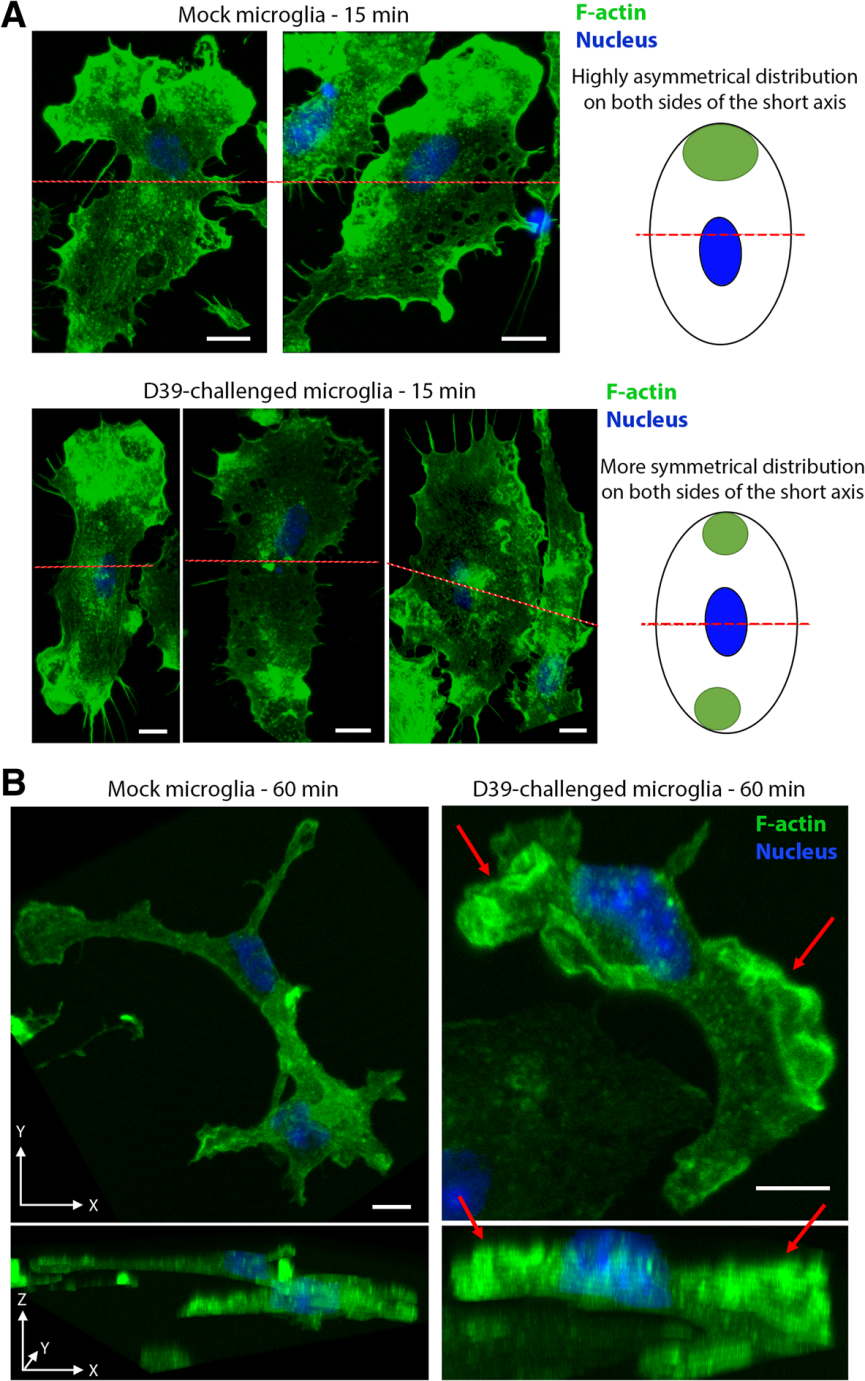
F-actin changes in microglia following exposure to pneumococci. F-actin staining with phalloidin-Alexa488 in microglia cells. **a** Asymmetrical (polarized) focal adhesion accumulation in mock-treated cells. The red lines represent the shorter axes of the cells, which should be approximately perpendicular to the direction of cell displacement. Shortly after microglia challenge with D39 lysates, focal adhesion clusters appeared on both sides of the shorter axes, disturbing polarization. **b** Sixty minutes after D39 challenge, microglia demonstrated multiple membrane ruffles (red arrows), clearly detectable in the *XY* (upper image) and in the *Z*-axis (lower panel). Scale bars, 10 μm

Next, we analyzed the distribution of F-actin (stable actin) in microglia. Eighty percent of the mock-treated cells demonstrated polarized phenotype with increased staining of accumulated actin at the cell bottom in one of the cell poles (focal adhesion clusters) (Fig. 6a). Fifteen minutes after challenge with D39 lysates, however, actin cell adhesion phenotype changed and > 60% of all microglia lost their polarized phenotype forming smaller, more symmetrically positioned focal adhesions clusters (Fig. 6a). Exposure of cells to lysates for 1 h led to the occurrence of massive membrane ruffles along the membrane in nearly all microglia (Fig. 6b), resembling the live imaging experiments in Fig. 5.

**Fig. 7:**
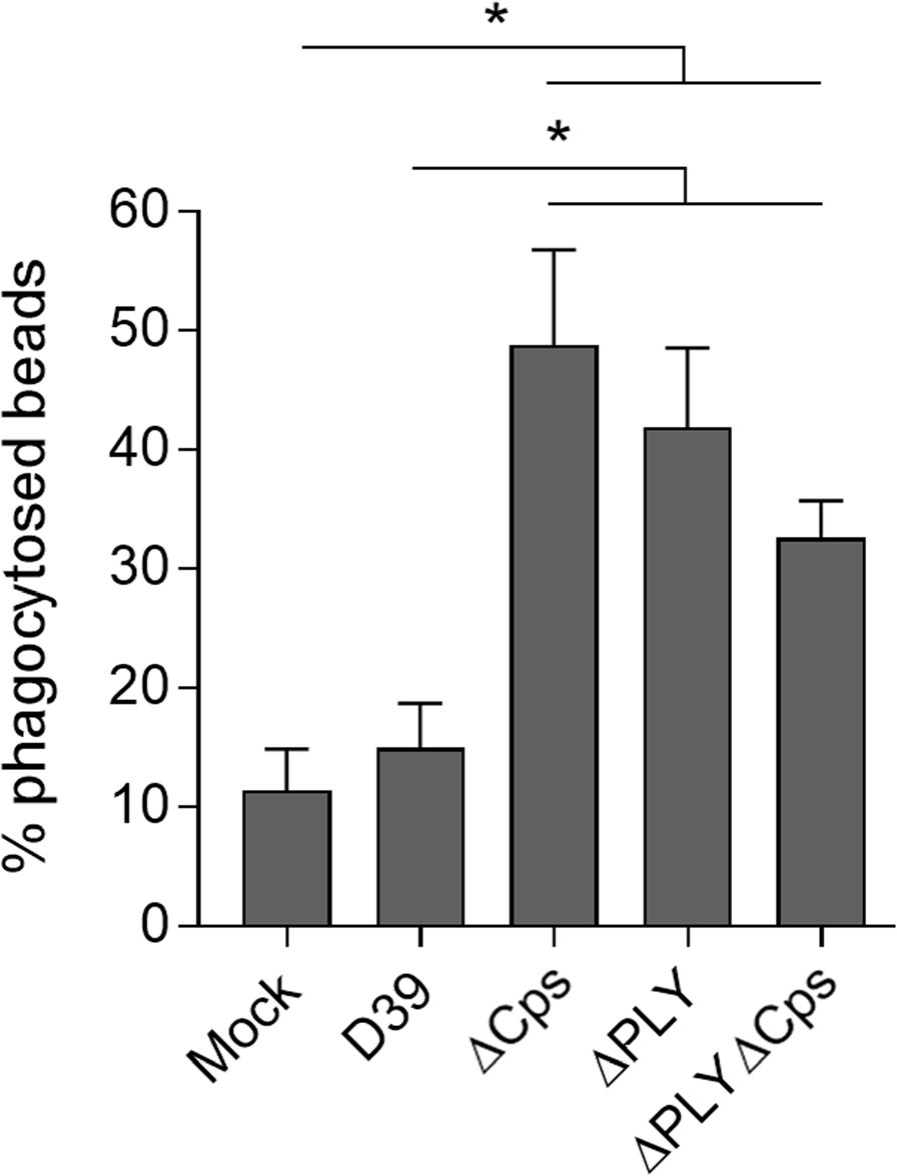
Phagocytosis enhancement by pneumococcal components. Analysis of phagocytosis by tracking beads in a mixed glial cell culture, demonstrating enhancement by single- (PLY or capsule) or double knockout (PLY and capsule) bacterial lysates, but not in the wild-type D39-treated cells. All values represent the mean ± SEM, *n* = 4 imaged fields from three independent experiments, *p < 0.05. In all bacterial lysis experiments, the lysate concentration was 20 million CFU/ml

Finally, we analyzed the ability of microglia to phagocytose 1 nm ovalbumin-coated fluorescent polystyrene beads. Briefly, non-phagocytosed beads outside of cells were stained with anti-ovalbumin antibody, while intracellular beads remained unstained. All beads were identified by their internal green fluorescence. We utilized mixed cultures with confluent monolayers of astrocytes. While bacterial lysates from wild-type D39 bacteria did not affect phagocytosis, the knockout mutants (ΔPLY, ΔCaps, and ΔPLYΔCaps) were capable of enhancing phagocytosis significantly (Fig. 7).

## Discussion

In this study, we demonstrated that microglia have a very agile response towards bacterial pathogens (such as *S. pneumoniae*), but key pneumococcal pathogenic factors such as bacterial capsules and pneumolysin substantially inhibit this effect shortly after exposure. While the bacterial capsule strongly inhibited basic microglial motility, in combination with pneumolysin, it also acted as a strong inhibitor of the taxis of microglia towards areas of damage, as represented by the cut surface interface of acute slices in our study. The effect of wild-type D39 pneumococcal lysates was microglial paralysis and inhibition of a directed response. This effect occurred within minutes after challenge, which differed from phagocytic migratory inhibition in bacterial infections due to factors, such as MIF and others, observed at 24 h and later in peripheral tissues [29].

Acute brain slices as a model of brain damage in meningitis recapitulate the changes observed in rat, mouse, and human diseases [30, 31]. Acute slices possess a line of damage (the cut plane), which resembles a necrotic interface. In this model system, bacterial products penetrate the tissue similarly as in real disease conditions, where multiple microabscesses are visualized throughout the cortex and the white matter [32]. In the initial phase of brain infection, bacteria colonize perivascular spaces and meninges, rapidly increasing their number (exponential or log-phase). After several hours, the growth reaches a stationary phase when the number of newly formed bacteria and lysed bacteria equalize [33]. The amount of free pneumolysin in CSF at this stage correlates well with the clinical course and disease prognosis of patients [34]. Lower molecular weight lytic products have much better penetration in the tissue than intact bacteria [8]; therefore, we logically focused on bacterial lysates as an experimental tool. For our experiments, we used between 5 and 10× lower dilutions than found in the CSF of patients—7 × 10^7^ CFU/ml (slices) and 2 × 10^7^ CFU/ml (dissociated cultures). In children with bacterial meningitis, the density of bacteria in the CSF can reach 4 × 10^9^ CFU/ml [35].

Microglia are the most reactive cell type in the brain, removing dying cells, limiting neural damage and inflammation (M2 phenotype), and/or inducing neurotoxicity and enhancing neuroinflammation (M1 phenotype) [36, 37]. Microglia are highly responsive towards invasive pathogens, and 24 h after the initiation of infection, they adopt an activated morphology [38]. Live imaging with fluorescently labeled phagocytes and bacteria in zebrafish has demonstrated a very early disease-limiting response of phagocytes when nonpathogenic PLY-deficient mutants multiply in the brain [39]. Conversely, D39 wild-type bacteria expand rapidly, and the phagocytic response remains limited. In this model, however, the differentiation between innate microglia and recruited circulating phagocytes (monocytes and neutrophils) is difficult. Nevertheless, it suggests that wild-type bacteria may indeed hamper phagocyte mobilization and recruitment early after disease onset. Similarly, our work demonstrates that double knockout mutants can substantially mobilize microglial recruitment towards bacterial lysates, most likely induced by other bacterial virulence factors (e.g., the peptidoglycans), but PLY and bacterial capsules inhibit this by impairing microglial motility. Microglia bear similarities to macrophages; they have common origin, functions, behaviors, and markers. Resembling macrophages, microglia are motile in tissues, and motility inhibition can have detrimental effects on infections. The mechanistic importance of phagocyte motility in infections has been demonstrated in an MMP-9 (matrix metallopeptidase 9)-knockout zebrafish model, where macrophages were shown to be less mobile in tissues, and the disease course is more severe [40]. Reduction of tissue phagocytes appears to be a targeted strategy of pathogens in infectious diseases, including pneumococcal sepsis and pneumonia [41, 42]. The inhibitory effect of PLY on microglia motility contrasts with its effects on other immune cell types such as the pro-chemotactic role in the recruitment of CD4 lymphocytes [43].

Microglial motility can be limited by inflammatory activators, such as lipopolysaccharides from Gram-negative bacteria [44]. Some studies caution this assumption, indicating several differences between animal strains in the responses of peritoneal macrophages to LPS when analyzing motility [45]. Endotoxin migratory inhibitory effects are often attributed to the secretion of the macrophage migration inhibitory factor (MIF). In our experiments, we observed the effects of lysates within 30–60 min after exposure to bacteria. We failed to detect different MIF levels between groups within a time window of 6 h. While bacterial factors from Gram-positive bacteria (cell wall-specific muramyl dipeptides) have also been shown to inhibit macrophage motility [46], these effects also require 20–24 h, while pneumococcal capsular effects are observed shortly after exposure, within minutes. In some of our experiments, we used a control treatment with CpG-DNA as a potent activator of TLR9-mediated innate microglial response to test whether innate immunity activation nonspecifically affects motility. This was not the case in live imaging motility experiments, suggesting that capsular effects were independent on the innate response activation status. TLR9 activation by CpG-DNA is a very potent innate immunity stimulator, but it is not the only receptor involved in innate responses and some other may affect microglial motility in a different way.

Microglia and astrocytes build a dynamic interaction unit in which one cell type influences the other. PLY acts on astrocytes, producing cell shape changes and glutamate release [8, 9, 47]. Astrocytes can also release ATP, which, after being degraded to adenosine, acts on microglia via adenosine receptors, initiating morphological changes [48, 49]. Therefore, it was essential to prove in pure microglial cultures that the effects of lysates were independent of astrocytes. The response of microglia to bacterial lysates from wild-type and mutant bacteria was very similar to that observed in mixed cultures, and it was rapid (within 60 min), confirming its astrocyte independence. In mixed cultures, however, microglia were substantially more motile due to the presence of an astrocyte monolayer. When isolated without astrocytes, microglia adhere stronger to the bottom of the dish and do not migrate as in mixed cultures.

**Fig. 8:**
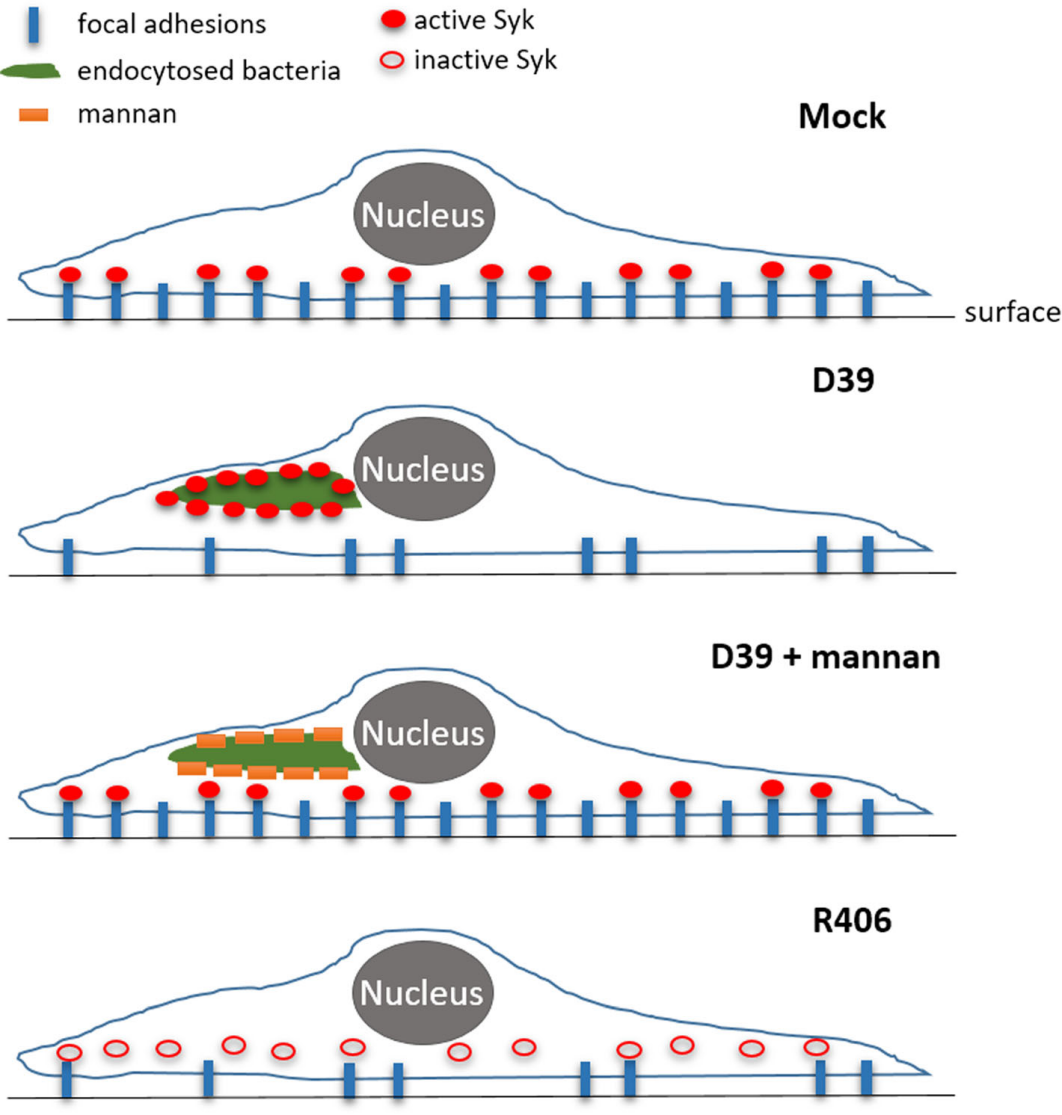
Schematic presentation of the suggested role of Syk in microglia as a factor in cell adhesion modulation by pneumococci. In resting microglia, the active phosphorylated Syk (pSyk) is associated with focal adhesions (as described in the literature). A sufficient number of focal adhesions is required in order to achieve cell motility. Upon exposure to pneumococcal lysates, the activity of Syk increases. Syk is recruited to phagosomes, but is depleted from focal adhesions; the number of focal adhesions drops, and motility is reduced. Upon exposure to mannan, the mannose receptor association with bacterial products is reduced, and the active Syk molecules remain associated with focal adhesions and are not recruited to phagosomes; microglial motility remains high. Upon application of R406, Syk is inhibited, including at the sites with focal adhesions, leading to reduction of their number and the loss of cell motility

The effect of PLY on the actin cytoskeleton, small GTPases, and microtubules has been demonstrated before [10–12]. The novel findings here regarding the role of the bacterial capsule are intriguing. The bacterial capsule plays an important role in the pathogenesis of infections and, specifically, in the virulence of *S. pneumoniae* as an inhibitor of opsonization and complement activation [50]. Capsules contain polysaccharides and are generally recognized by pattern recognition receptors [51]. Pneumococci are recognized by the Mincle receptor [52] and the mannose receptor [26]. Mannose receptors on olfactory ensheathing cells and Schwann cells are used by *S. pneumoniae* as invasion anchors [53]. The mannose receptor can be blocked by mannan [54]. Mannan is a mannose polysaccharide found in plants, fungi, and yeast cells. In our experimental setup, it influenced the capsule-dependent motility of microglia. The mannose receptor can initiate inflammatory factor release but can also be involved in cytoskeletal remodeling, especially during the early steps of phagocytosis [55, 56]. The changes in the Syk phosphorylation and its subcellular distribution after D39 exposure and their restoration after mannan pretreatment logically suggested a possible involvement of Syk in the observed motility effects. Thus, it was surprising that pharmacological inhibition of Syk did not recover the inhibited motility of microglia, but rather the opposite—R406 by itself diminished microglial motility and formed long filopodia-like tentacles, strongly resembling the phenotype, initiated by D39 lysates. Syk is a kinase with multiple roles in immune cells; many of its effects depend on the exact compartment where its activation takes place. In cell motility, activated Syk is important locally for the formation of focal adhesions and substrate attachment of leukocytes [57]. Recruitment of activated Syk to subcellular structures containing bacterial products (presumably endo- and phagosomes) can secondarily deplete pSyk at focal adhesion sites and in the end produce an identical phenotype as the pharmacological inhibition of the kinase. A summarized overview of this concept is presented in Fig. 8.

Seemingly controversial is our finding that in bacterial knockout experiments the bacterial capsules, but not PLY, affected motility, but in recombinant PLY exposure experiments, PLY did inhibit microglial motility. Bacteria are complex pathogens and their pathogenic factors may interact in a manner, which is not consistent with the properties of isolated ones. A recent finding confirms that PLY binds the mannose receptor in immune cells and initiates inhibitory receptor effects, while bacterial capsules activate the receptor [58]. Furthermore, differences in PLY levels between bacterial lysates and recombinant toxin may play a role as well. These results indicate the necessity to use complex systems containing complete bacteria rather than isolated factors in infectology studies.

Cell migration starts by traction at focal adhesions (points of interaction between the cells and the extracellular matrix) at the leading edge of cells, typically in areas of lamellipodia formation [59]. The early changes in F-actin after D39 challenge suggested altered adhesion and loss of polarity and subsequent loss of directionality as a possible reason for migration block. In migrating cells, asymmetric adhesion represents a critical biophysical requirement for directional displacement and exactly this changed in microglia after exposure to D39 [60]. The occurrence of massive membrane ruffles 1 h after bacterial challenge hinted at ongoing actin modifications. These actin changes were consistent with our knowledge of the role of PLY as an actin- and GTPase-modulating factor as well [10, 11].

We observed a similar effect of bacterial capsules in experiments with both serotype 2 (the D39 strain) and serotype 6B pneumococci, confirming that the effects were not limited to only one serotype. The exact magnitude of capsular effects in both stains we used, however, differed. Many factors can contribute to this difference—capsular properties and capsular amounts, but also differences in the expression levels of pneumolysin and other pathogenic elements that can modulate the capsular effects on microglia as well.

## Conclusions

Our findings suggest that pneumococci utilize PLY and capsule components for very early microglial migratory inhibition before the expression of MIF and other pro-inflammatory factors start. The fact that such inhibition occurs so early signifies the importance of phagocyte inhibition by bacteria throughout the stages of disease pathogenesis. The involvement of the bacterial capsule in these effects suggests a more significant role in pathogenic processes and requires further studies.

## Additional files

Additional file 1: **Figure S1.** Verification of the purity of microglial preparations by immunocytochemistry with isolectin-B4 (red) and nuclear counterstaining with DAPI (cyan). All isolated cells are microglia. Scale bar: 40 μm. (TIF 14292 kb)

Additional file 2: **Figure S2.** Comparison of Iba1-positive cells within the top 20 μm of the cut surface of acute slices immediately after sectioning and 6 h later, demonstrating increased microglial taxis towards the area of tissue damage. Microglia demonstrates rounded activated morphology after 6 h *versus* the resting stellate morphology immediately after sectioning. Scale bar: 30 μm. All values represent the mean ± SEM, *n* = 3 slices. (TIF 6132 kb)

Additional file 3: **Movie M1.** 3D (50 μm depth) reconstruction movie of Iba1-positive microglial cells clustering towards the surface of mock-treated acute brain slices. (AVI 4160 kb)

Additional file 4: **Movie M2**. 3D (50 μm depth) reconstruction movie of Iba1-positive microglial cells clustering towards the surface of dual bacterial mutant lysate-treated (∆PLY∆Cps) acute brain slices. (AVI 5508 kb)

Additional file 5: **Movie M3**. 3D (50 μm depth) reconstruction movie of Iba1-positive microglial cells clustering towards the surface of D39 lysate-treated acute brain slices. (AVI 3633 kb)

Additional file 6: **Movie M4.** Time-lapse imaging of mock-treated mixed glial cell cultures for 60 min (30 s intervals), demonstrating relatively stationary astrocytes and highly dynamic microglia. (AVI 2739 kb)

Additional file 7: **Movie M5**. Time-lapse imaging of mock-treated mixed glial cell cultures for 60 min (30 s intervals) overlaid with an Iba1-immunostained image shortly after the time-lapse, demonstrating complete overlay between Iba1-positive cells and motile cells. (AVI 2893 kb)

Additional file 8: **Movie M6**. Time-lapse imaging of the long filopodia-like membrane extensions formed in mixed glial cultures by lysates from double mutant (∆PLY∆Cps) bacteria. The interval between two frames is 4 min. (AVI 231 kb)

Additional file 9: **Movie M7**. Time-lapse imaging of isolated mock-treated microglia. The interval between two frames is 30 s. (AVI 24144 kb)

Additional file 10: **Movie M8**. Time-lapse imaging of isolated microglia treated with ∆PLY∆Cps lysate. The interval between two frames is 30 s. (AVI 22852 kb)

Additional file 11: **Movie M9**. Time-lapse imaging of isolated microglia treated with ∆Cps lysate. The interval between two frames is 30 s. (AVI 7464 kb)

Additional file 12: **Movie M10**. Time-lapse imaging of isolated microglia treated with ∆PLY lysate. The interval between two frames is 30 s. (AVI 12032 kb)

Additional file 13: **Movie M11**. Time-lapse imaging of isolated microglia treated with D39 lysate. The interval between two frames is 30 s. (AVI 12537 kb)

## Abbreviations

∆Cps: Capsule-deficient D39 *S. pneumoniae* strain mutant
∆PLY: Pneumolysin-deficient D39 *S. pneumoniae* strain mutant
∆PLY∆Cps: Double pneumolysin- and capsule-deficient D39 *S. pneumoniae* strain mutant
106.66 Janus: Capsule-deficient (Janus-cassette containing) strain 106.66 of the 6B serotype of *S. pneumoniae*
106.66: Strain 106.66 of the serotype 6B *S. pneumoniae*
aCSF: Artificial cerebrospinal fluid
ATP: Adenosine triphosphate
BSA: Bovine serum albumin
CFU: Colony-forming unit
CLR: C-type lectin receptor
CpG-DNA: Unmethylated cytosine- and guanine-rich (CG-rich) DNA motifs, typical for bacterial DNA
Cps: Capsule
DAPI: 4,6-Diamidin-2-phenylindol
EDTA: Ethylenediaminetetraacetic acid
LAL: Limulus amebocyte lysate
LDH: Lactate dehydrogenase
MES: 2-(N-morpholino)ethanesulfonic acid
MIF: Macrophage migration inhibitory factor
MMP: Matrix metallopeptidase
PBS: Phosphate-buffered saline
PI: Propidium iodide
PLO: Poly-l-ornithine
PLY: Pneumolysin
pSyk: Phosphorylated Syk at positions Tyr525 and Tyr526
SOCS1: Suppressor of cytokine signaling 1
Syk: Spleen tyrosine kinase
TLR9: Toll-like receptor 9
