# Supplementary figures and images for: Pneumolysin and the bacterial capsule of Streptococcus pneumoniae cooperatively inhibit taxis and motility of microglia
Source: J Neuroinflammation. 2019 May 18;16:105. doi: 10.1186/s12974-019-1491-7 (PMC6525981; doi:10.1186/s12974-019-1491-7)

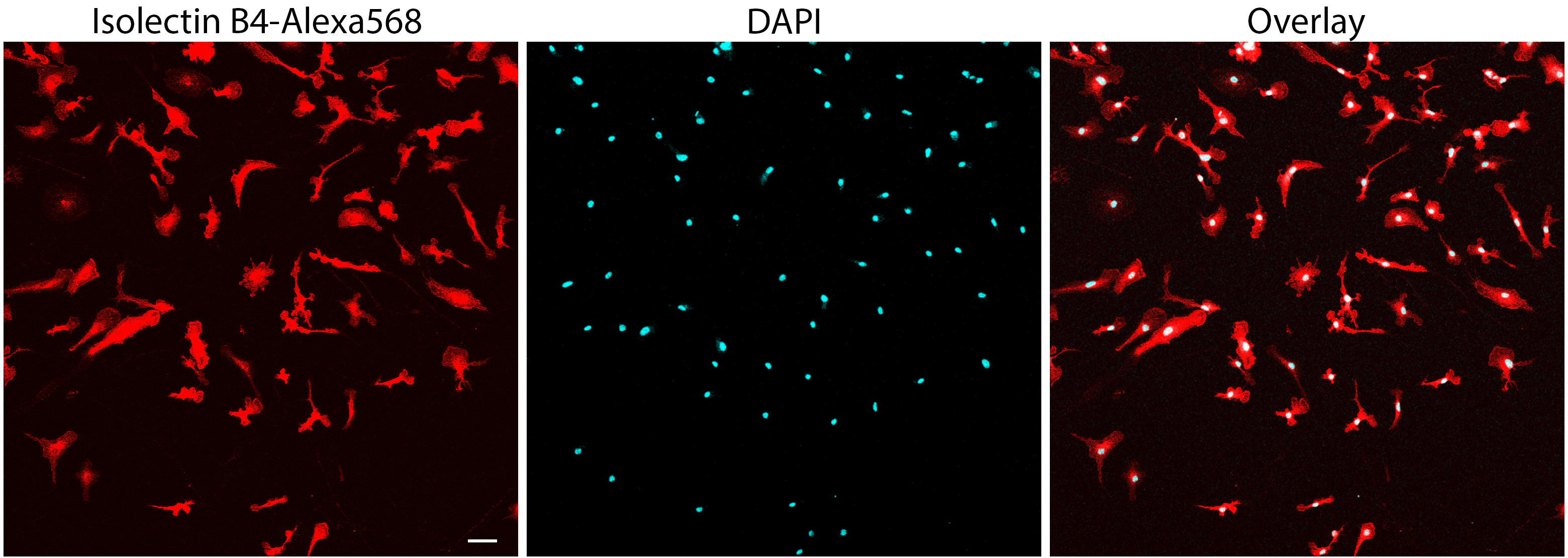

Supplement: Supplementary file 1 — Figure S1. Verification of the purity of microglial preparations by immunocytochemistry with isolectin-B4 (red) and nuclear counterstaining with DAPI (cyan). All isolated cells are microglia. Scale bar: 40 μm. (TIF 14292 kb) [file 12974_2019_1491_MOESM1_ESM.tif]

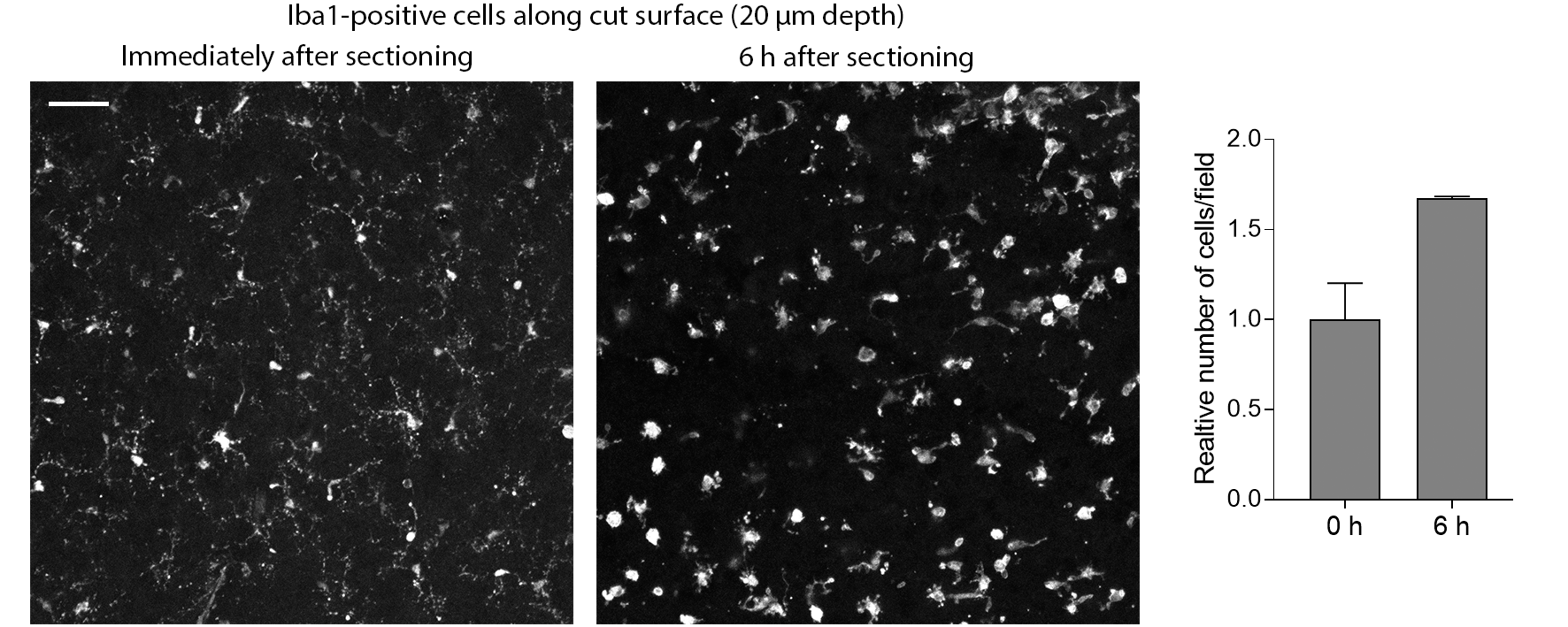

Supplement: Supplementary file 2 — Figure S2. Comparison of Iba1-positive cells within the top 20 μm of the cut surface of acute slices immediately after sectioning and 6 h later, demonstrating increased microglial taxis towards the area of tissue damage. Microglia demonstrates rounded activated morphology after 6 h versus the resting stellate morphology immediately after sectioning. Scale bar: 30 μm. All values represent the mean ± SEM, n = 3 slices. (TIF 6132 kb) [file 12974_2019_1491_MOESM2_ESM.tif]
